# Acute Liver Failure With Severe Lactic Acidosis Secondary to Infiltrative Diffuse Large B-Cell Lymphoma: An Imaging-Negative Presentation

**DOI:** 10.7759/cureus.10110

**Published:** 2020-08-29

**Authors:** Andre Hafner, David B Eaton

**Affiliations:** 1 Internal Medicine, University of South Dakota Sanford School of Medicine, Rapid City, USA; 2 Internal Medicine, Monument Health, Rapid City, USA

**Keywords:** dlbcl, lactic acidosis, acute liver failure, secondary hepatic lymphoma

## Abstract

Liver involvement by non-Hodgkin’s lymphoma is common in late stage disease but rarely results in severe hepatic dysfunction. Here, we discuss a case of acute liver failure (ALF) with severe lactic acidosis in a 75-year-old female with diffuse large B-cell lymphoma (DLBCL). The patient was admitted with nausea, fevers, and mild acidosis. Although radiographic imaging did not demonstrate any liver abnormality, the patient soon developed signs of ALF along with severe lactic acidosis. Despite initiation of chemotherapy, the patient deteriorated quickly and was ultimately put on comfort measures. This case highlights an uncommon manifestation of DLBCL and suggests that an accelerated timeline for beginning chemotherapy may be warranted in patients with high clinical suspicion of secondary hepatic lymphoma.

## Introduction

Diffuse large B-cell lymphoma (DLBCL) is the most common subtype of non-Hodgkin's lymphoma (NHL), which is the most common hematologic malignancy worldwide [1]. Despite this relative frequency, DLBCL should be recognized as a heterogeneous grouping of various subtypes that share a common morphology but differ in terms of prognosis and treatment response [2]. In most cases, initial symptoms are nonspecific and patients frequently present with late stage disease. Signs and symptoms can be focal or systemic and reflect both metabolic derangements and manifestations of underlying organ damage [3]. Although liver involvement is estimated to occur in 16%-46% of patients with DLBCL, it very rarely leads to acute liver failure (ALF) [4]. In the majority of cases, liver involvement represents extranodal extension of the disease rather than a primary hepatic lymphoma [5].

Our case represents an example of malignancy-associated ALF with associated lactic acidosis and liver function derangements. Of note, the patient did not display hepatomegaly and did not show any signs of liver involvement on ultrasound or CT imaging prior to liver failure. Given the occult presentation of hepatic infiltration in this patient, we believe this case represents a valuable addition to the literature and emphasizes the importance of strong clinical suspicion in the absence of radiographic findings.

## Case presentation

A 75-year-old female with generalized abdominal pain and fatigue presented to the emergency room after lab studies at an urgent care showed acute kidney injury. Past medical history was significant for hypertension, hypercholesterolemia, restless leg syndrome, and peptic ulcer disease. Home medications included atorvastatin, losartan-hydrochlorothiazide, pramipexole, and bupropion. Review of systems was positive for 12 pounds of weight loss over the previous two weeks, subjective fever, and decreased appetite.

On presentation, vital signs showed heart rate of 96 beats/minute, blood pressure of 121/74 mmHg, a temperature of 37°C, and oxygen saturation of 95% on room air. Physical exam was remarkable for diffuse abdominal tenderness without guarding. EKG showed sinus rhythm and no ST elevation or arrhythmias. A CT scan of the abdomen and pelvis without IV contrast was significant for extensive lymphadenopathy in the retroperitoneum and the para-esophageal area near the splenic hilum highly suspicious for lymphoma. Aggressive IV fluids were initiated, and the patient’s condition improved to the point where she was discharged after two days with close follow-up and scheduled lymph node biopsy.

**Figure 1:**
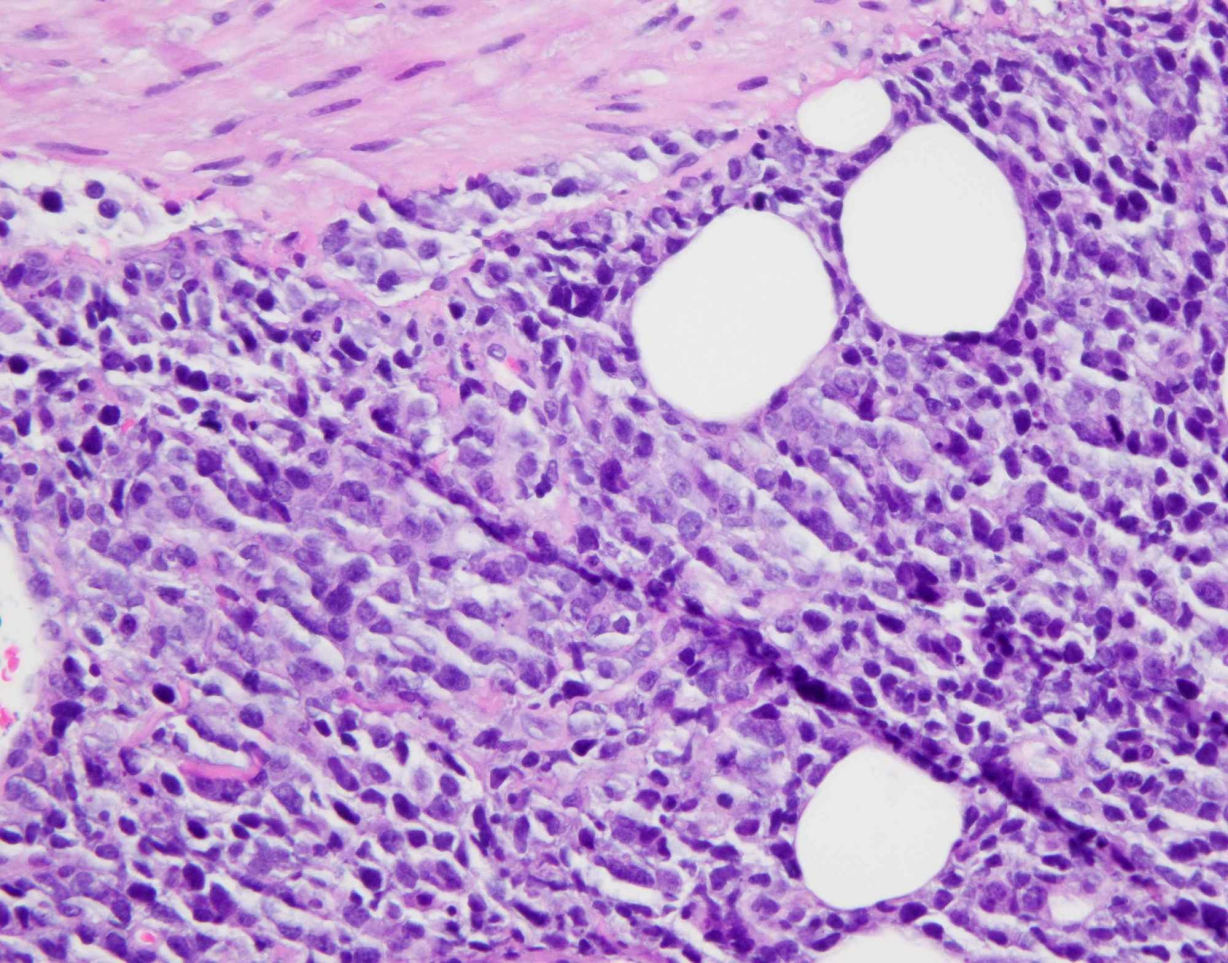
Retroperitoneal lymph node biopsy showing diffuse proliferation of large lymphocytes (hematoxylin-eosin staining; H&E)

The patient’s condition soon worsened, however, and she returned to the ER two days later with recurrent epigastric and right upper quadrant (RUQ) abdominal pain, vomiting, diarrhea, and chest pain. Laboratory studies showed an elevated anion gap of 14 mmol/L and a mildly elevated aspartate aminotransferase (ALT) of 84 U/L. The patient was admitted and started on IV fluids. Blood cultures were drawn. A GI pathogen panel was collected and was found to be negative. Serology tests for hepatitis A, B, and C were negative. Over the following three days, the patient continued to experience abdominal pain, nausea, weakness, and fevers. The mild acidosis initially resolved, but the liver enzymes continued to rise. The patient developed hypoglycemia and worsening thrombocytopenia. CT-guided para-aortic lymph node biopsy was performed on day 4, and pathology confirmed stage IV NHL, diffuse large B cell type (Figure 1).

On hospital day 5, the patient was transferred to the ICU due to the onset of progressive metabolic acidosis and dyspnea concerning for sepsis or ischemic bowel. In addition, the patient’s worsening confusion, hypoglycemia, and coagulopathy indicated possible ALF (Table 1). 

**Table 1:** Laboratory data showing deterioration of liver function WBC, white cell count; Hb, hemoglobin; ALT, alanine transaminase; AST, aspartate transaminase; ALP, alkaline phosphatase; INR, international normalized ratio; aPTT, activated partial thromboplastin time

Labs	Day 1 (admission)	Day 5	Day 7
WBC (10^3^/µL)	5.2	6.3	3.3
Hb (g/dL)	10.1	10.1	7.2
Platelets (10^3^/µL)	97	93	24
AST (U/L)	84	453	3,219
ALT (U/L)	47	109	618
ALP (U/L)	148	159	285
Total bilirubin (mg/dL)	1.12	1.89	2.76
Glucose (mg/dL)	87	70	165
Prothrombin time (s)	--	22.6	26.5
INR	--	2.0	2.3
aPTT (s)	--	33.2	30
Fibrinogen (mg/dL)	--	--	163
Anion gap (mmol/L)	14	30	17
Lactate (mmol/L)	--	17.5	8.0
pH	--	7.09	7.40
HCO_3_- (mmol/L)	20	13	26

**Figure 2:**
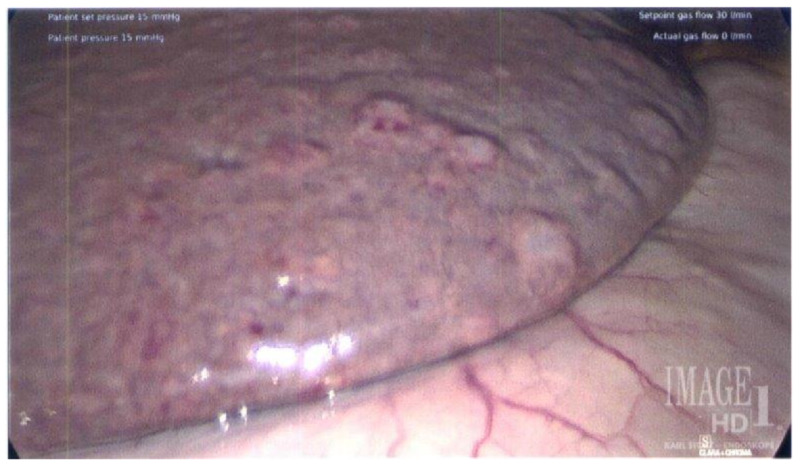
Intraoperative image of the liver showing micro to moderate nodularity

The patient was started on a bicarb drip and empiric antibiotics. Later that day, the patient underwent a diagnostic laparoscopy followed by laparoscopic cholecystectomy and multiple liver biopsies. During the surgery, the liver was noted to look grossly abnormal showing micro to moderate nodularity (Figure 2). There was no evidence of bowel ischemia, perforation, or inflammatory changes. Following surgery, the patient developed hemodynamic instability, most likely due to severe acidosis and liver failure. The patient was started on vasopressors and remained intubated with mechanical ventilation. 

It was assumed at this point that the liver dysfunction was related to malignant hepatic infiltration. Chemotherapy with rituximab, cyclophosphamide, doxorubicin, vincristine, and prednisone (R-CHOP) along with rasburicase was initiated on day 6 with appropriate dose reductions for renal and liver failure. During the following two days, the patient suffered seizures and worsening uremia. Platelet count worsened acutely, and this was attributed to underlying hepatic failure and possible disseminated intravascular coagulation rather than drug toxicity. The patient was started on continuous renal replacement therapy on day 7 due to oliguria and progressive renal failure.

Over the following four days, the patient continued to deteriorate clinically. Blood cultures did not show growth and the patient was transitioned off antibiotics. Multiple transfusions of fresh-frozen plasma (FFP) and platelets were required due to thrombocytopenia and coagulopathy. Although there was initial success in controlling the patient’s metabolic lactic acidosis and hemodynamic status, she remained unresponsive despite being off all sedation. Ultimately, the family decided to transition the patient to comfort care measures on day 11 and the patient expired two days later.

## Discussion

ALF is an uncommon diagnosis that is largely associated with viral hepatitis, toxic ingestion, or drug reactions [6]. It is defined by findings of coagulopathy (international normalized ratio [INR] > 1.5) and encephalopathy in a patient without pre-existing liver disease and an illness lasting less than 26 weeks. Malignant infiltration of the liver is a rare cause of ALF but may be more common than previously reported. Although one frequently cited single center study completed in 1998 reported a incidence of 0.44% [7], two more recent multicenter studies completed in 2012 and 2015 report an incidence of 1.4% [8] and 1.8% [9], respectively. ALF in this setting carries a particularly poor prognosis with median survival from time of admission being reported as low as six days.

Determining the etiology of ALF is critical for guiding treatment but can be especially challenging in cases of malignant infiltrative disease. Symptoms and laboratory findings of ALF are nonspecific; however, a complete history should give some indication of possible malignant etiology, such as a history of B-symptoms or unexplained weight loss [10]. Physical exam may show lymphadenopathy or hepatosplenomegaly. Initial serology should exclude common viral etiologies. Most cases of hepatic lymphoma are secondary rather than primary to the liver; therefore, CT imaging should demonstrate enlarged lymph nodes and delineate their pathologic extent. However, in patients with NHL ultrasound and CT findings can be unreliable in demonstrating liver involvement [11,12]. This is particularly challenging in patients with a diffuse pattern of liver involvement and nodules less than 1 cm in size who may only have findings of hepatomegaly or no liver findings at all. Further characterization of the malignancy with imaging should involve a combined positron emission tomography/CT (PET/CT), which has been shown to have a sensitivity approaching 100% for splenic involvement of lymphoma and is vital for determining the extranodal extent of disease [11]. Further imaging with MRI is not part of the standard protocol for suspected hepatic lymphomas, but may be considered to further characterize an incidental liver finding in the absence of clinical symptoms. A PubMed search involving the keywords secondary hepatic lymphoma (SHL) and NHL did not yield any studies that investigated the sensitivity or specificity of MRI in the setting of suspected hepatic lymphoma.

**Figure 3:**
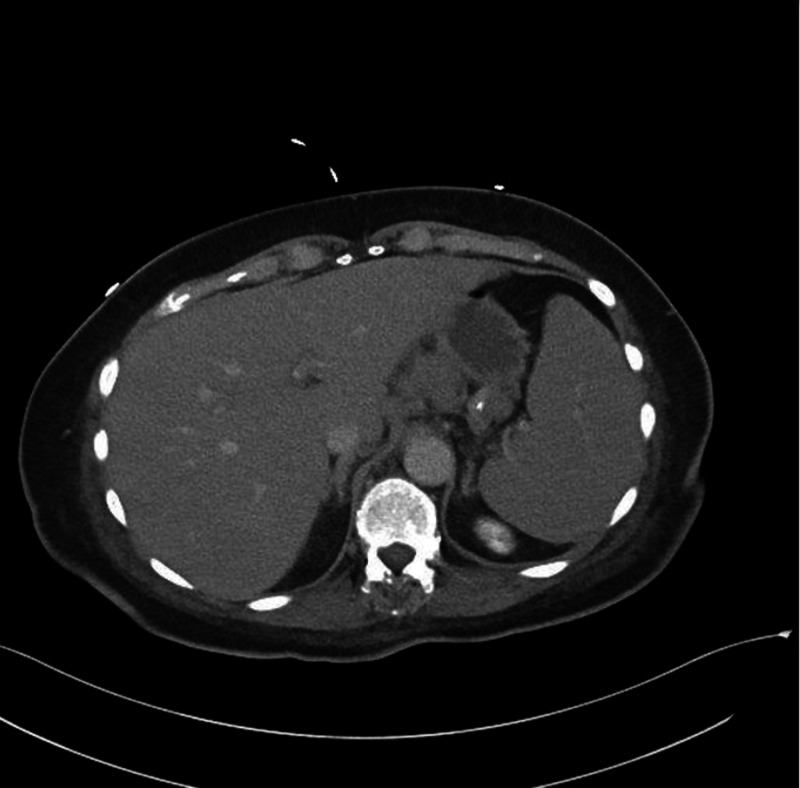
CT with IV contrast reveals normal appearing liver and moderate splenomegaly

**Figure 4:**
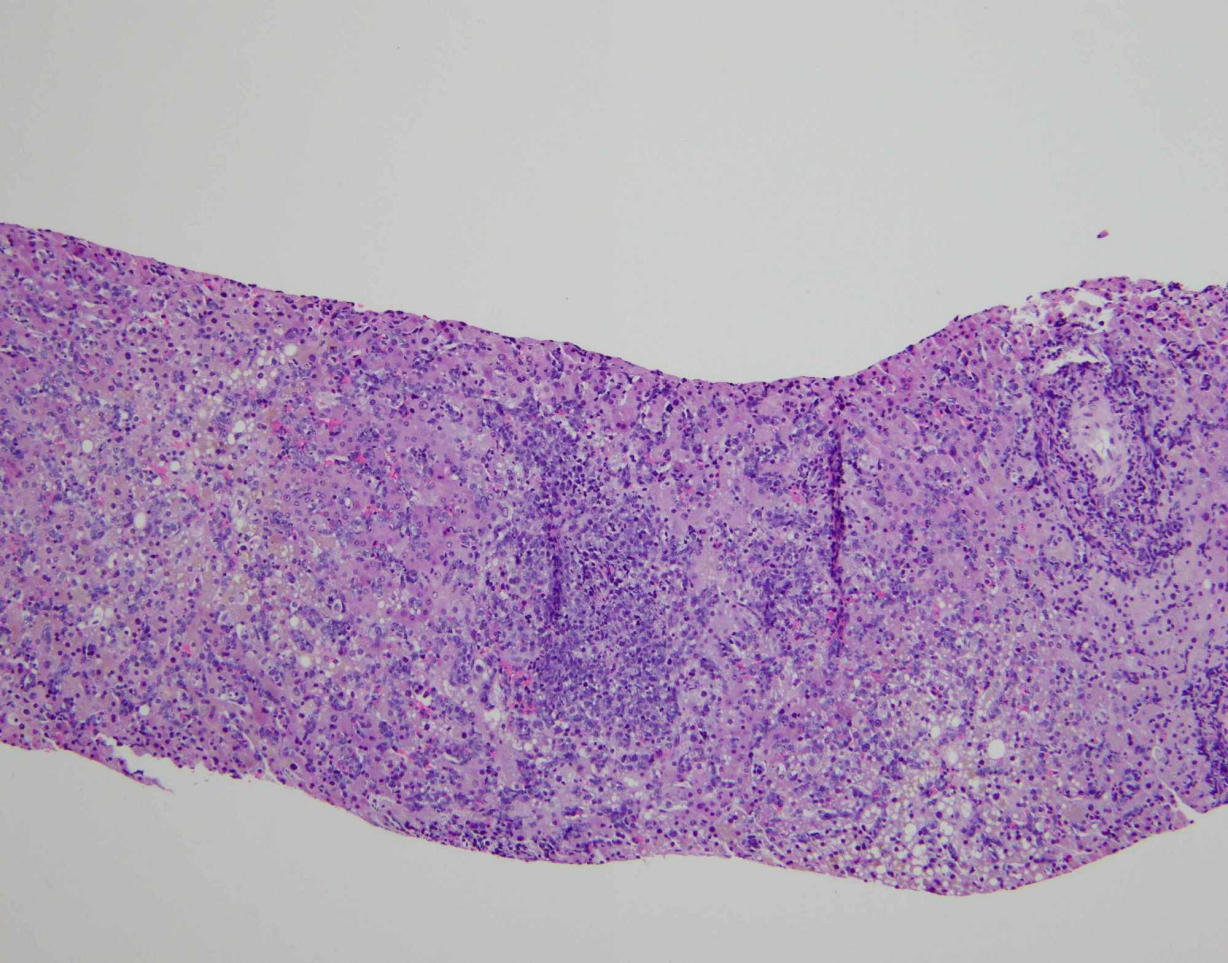
A needle core biopsy of the liver showing diffuse infiltration by tumor cells

In our case, CT of the abdomen with contrast did not show any liver abnormalities (Figure 3). Although liver involvement was strongly suspected due to clinical and laboratory signs of ALF, it was only during exploratory surgery that the liver was noted to look grossly abnormal. PET/CT was delayed indefinitely due to the acuity of the patient’s condition and biopsy later confirmed diffuse malignant infiltration (Figure 4).

An important consideration for the differential diagnosis in this patient relates to the finding of severe lactic acidosis. This ominous sign has been shown to be associated with a poor prognosis in patients with lymphoma [13]. The underlying etiology of lactic acidosis can be multifactorial and is often elusive. Infectious, metabolic, and ischemic causes must all be considered. Sepsis is a major concern in patients with a hematologic malignancy and is associated with varying degrees of liver dysfunction. Metabolic effects of lymphoma can also elevate lactic acid due to either increased glycolysis in cancer cells via the Warburg effect or due to tumors that outgrow their blood supply [14].

In our case, multiple blood cultures were negative both before and after the onset of lactic acidosis and our patient had no response to broad-spectrum antibiotics. No source of infection was identified. Therefore, it appears that the lactic acidosis was due to a combination of metabolic effects and localized ischemia. Indeed, one mechanism of liver damage in lymphoma is extensive infiltration of the sinusoids and hepatic vasculature leading to diffuse hepatic necrosis [15]. Finally, decreased lactate clearance due to impaired liver and renal function likely contributed to the acidosis [13].

Due to the infrequency of ALF secondary to lymphoma, there are no definite guidelines for treatment. SHL is characteristic of late stage disease, and the International Prognostic Index (IPI) indicates an age-adjusted overall survival of 21% in a high risk patient above 80 years of age [1]. Thus, earlier diagnosis and treatment should be the primary goal. A search of the literature found five cases of survival in patients with ALF associated with extranodal DLBCL [16-20]. All five of these patients received a variation of standard therapy based off the R-CHOP regimen, but specific treatment differed significantly from one patient to the next. In our case, R-CHOP with dose reductions and rasburicase was initiated on hospital day 7 but was unable to reverse the course of the patient’s disease. 

## Conclusions

SHL is a rare but important cause of ALF that carries a very poor prognosis. Confirmatory biopsy can be difficult to obtain in the setting of ALF, and the underlying etiology is not always forthcoming on imaging. Our case review suggests that even in the absence of hepatomegaly or radiographic abnormalities, a constellation of findings including lactic acidosis, coagulopathy, and hypoglycemia should raise strong suspicion for secondary hepatic involvement leading to ALF. Although there is no standard treatment for ALF in the setting of SHL, early initiation of chemotherapeutic agents has been demonstrated to result in long-term survival in a minority of patients.
